# A longitudinal study of multicultural curriculum in medical education

**DOI:** 10.5116/ijme.52ec.d075

**Published:** 2014-02-22

**Authors:** Mary L. Zanetti, An Dinh, Laura Hunter, Michael A. Godkin, Warren Ferguson

**Affiliations:** 1 Office of Institutional Research, Evaluation and Assessment, University of Massachusetts Medical School, USA; 2 Department of Family Medicine and Community Health, University of Massachusetts Medical School, USA

**Keywords:** Cultural humility, cultural competence, medical education, and multicultural curriculum

## Abstract

**Objectives:**

To evaluate impact a multicultural interclerkship had on students’ perception of knowledge, interview skills, and empathy towards serving culturally diverse populations and role student demographics played in learning.

**Methods:**

Data extracted from students’ self-reported course evaluations and pre/post questionnaires during multiculturalism interclerkship across 11 academic years. Inquired students’ opinion about four areas: effectiveness, small group leaders, usefulness, and overall experience. Subscale and item ratings were compared using trend tests including multivariate analyses.

**Results:**

During studied years, 883 students completed course evaluation with high overall mean rating of 3.08 (SD=0.45) and subscale mean scores ranging from 3.03 to 3.30. Trends in three of four subscales demonstrated clear uptrend (p<0.0001). Positive correlations between ratings of leaders and “usefulness” were observed (p<0.0001). Pre/post matched dataset (n=967) indicated majority of items (19/23) had statistically significant higher post interclerkship ratings compared to pre scores with nine of 19 having statistically significant magnitudes of change. Questionnaire had high overall reliability (Cronbach alpha=0.8), and item-to-group correlations ranged from 0.40 to 0.68 (p <0.0001).

**Conclusions:**

By increasing students’ exposure and interaction with diverse patients, their knowledge, attitude, and skills were increased and expanded in positive manner. These findings might inform those who are interested in enhancing this important competence. This is especially true given increasing scrutiny this global topic is receiving within and across healthcare professions around the world.

## Introduction

Increasing health disparities across the globe has led to a wide array of governmental and educational initiatives. Each initiative is responding to the need to better prepare students and healthcare providers to promote a more culturally competent healthcare system. In 2009, the Accreditation Council for Continuing Medical Education (ACCME) in the United States announced a new cultural and linguistic competence (CLC) standard “to assure that cultural and linguistic understanding is effectively integrated into physician education”.^1^ Additionally, in 2011 the Health and Human Service (HHS) agency in the United States defined clinical cultural competence [now often referred to as cultural humility in many research journal publications] as “the ability of health care professionals to communicate with and effectively provide high-quality care to patients from diverse sociocultural backgrounds.”^2^Most recently in July 2012, a joint expert panel of educators was convened by the Association of American Medical Colleges and the Association of Schools in Public Health.^3^ The panel was charged with identifying a set of competencies appropriate for learners in both disciplines to prepare culturally competent practitioners. The joint panel built upon existing research including the 2003 Liaison Committee on Medical Education (LCME) addition of a “cultural competence” component to its accreditation standards requiring US and Canadian medical schools to ensure its students and faculty are able to demonstrate an understanding of the role culture has on patients’ perceptions of illness, and treatment, and overall healthcare delivery.^3^^-^^5^ Regardless of the specific definition of cultural competence, it is widely agreed that this far-reaching topic needs increased attention within healthcare curriculum today throughout the world.The outlined initiatives have contributed to a more comprehensive view of the role culture plays in patient and physician interaction, compliance with medical treatment, and overall successful healthcare delivery. In other words, physicians and learners are now being asked to better understand and utilize the role culture plays in daily interaction with all patients as this will improve the manner in which healthcare is delivered and received by patients in all countries. Eliminating healthcare disparities requires numerous strategies and interventions over a long time frame,^1^ and medical schools across the globe can do their part by evaluating and assessing the strength of their “cultural competence” curriculum to ensure it is current and effective. On-going evaluation of the effectiveness of cultural competence curriculum in all medical schools is essential because it is important that students receive early and often exposure to the knowledge, skills, and attitudes connected to cultural competence in their education. Frequent exposure might then feed into the realization that there is a need for lifelong learning as graduates move into practice as future physicians anywhere in the world.Recent medical education research studies have found female students and older students to be more empathic; additionally, another study suggested rural upbringing and training may not be sufficient to encourage healthcare professionals to practice in rural settings.^6^^-^^9^ Society needs competent physicians who are sensitive to ethnic, cultural and gender diversity within communities. ^10^ This introspective process may help reduce racial and ethnic disparities in health and healthcare. ^1^^,^^11^^-^^16^ Four previous studies involving the fourth author resulted in a wide range of findings connected to the effectiveness of a global multicultural pathway.^17^^-^^20^ While some studies suggested no effect from multicultural education,^10^^,^^11^ the most recent of the four studies found clear and distinct differences between two groups of students indicating the root of increased confidence in cultural competence may have developed from exposure to a specific multicultural pathway program with future research warranted to determine the impact of other similar experiences at the studied institution. All of these summarized research findings clearly indicate that a multi-modal approach to educating medical students about cultural competence and disparities in healthcare may be beneficial.We did not find any published studies analyzing a multicultural curriculum at a medical institution over a long period of time. This study represents a continued research effort focused on analyses of 11 years of data associated with a specific multicultural curriculum in a medical school; thereby, providing academic leaders with the ability to make thoughtful decisions about the effectiveness of a specific multicultural curriculum. More specifically, the purpose of this study is to evaluate the impact a multicultural interclerkship had on student knowledge, interview skills, and empathy towards serving culturally diverse populations and the role student demographics played in their learning. Additionally, we present results from student course evaluations to illustrate student perceptions of the interclerkship.

## Methods

### Study design

We conducted a quantitative observation study which included cross sectional design using course evaluation and a pre/post intervention design using questionnaires to evaluate students’ knowledge, empathy, and interview skills over 11 academic years (AY) from AY 2000/2001 to AY 2010/2011 at the University of Massachusetts Medical School (UMMS) in the United States.

### Participants and sample size

Study participants included all third-year medical students at the UMMS who were required to participate and complete evaluations in a multicultural interclerkship, one of several required one-day courses between clerkships, designed to present curriculum topics that cross all specialties. The interclerkship is designed to teach narrative interviewing techniques followed by skills practice as a means to better understanding patients’ health beliefs and practices, histories of immigration, and experiences of seeking and obtaining healthcare. Initial design of the curriculum included standardized patients for small group practice. However, students and faculty both concluded that this was an inappropriate use of standardized patients, missing an opportunity for authentic discovery and risking stereotyping. Subsequently, community culture teachers, who are health and human services professionals from cultures reflective of the local population, were employed to make the small group experience more meaningful in response to the intial student feedback. Community culture teachers are trained to share their own personal stories as well as their perceptions of the experiences of patients whom they serve. In addition to small group practice sessions, students engage in workshops on a variety of topics germane to serving diverse communities.

### Data collection procedures

Data for these analyses were extracted from students’ self-reported, Likert-scale course evaluations and pre/post questionnaires routinely collected during each year’s multiculturalism interclerkship from AY2000-01 through 2010-11. Although course evaluation forms varied slightly across these eleven years, a common group of twenty quantitative questions were identified for analyses. These questions inquired students’ opinion about four areas: the effectiveness of the workshop (three 4-point scaled questions), evaluations of the small group leaders (three 4-point scaled questions), the usefulness of this interclerkship (seven 4-point scaled questions), and the overall experience (seven 5-point scaled questions). Means of section ratings and individual item ratings were calculated and compared over the years using trend tests.Pre and post questionnaires consisted of 23 identical 4-point scaled items (strongly disagree, disagree, agree, strongly agree) seeking participants’ ratings of their empathy (six items), knowledge (eight items), and interview skills (nine items) while working with culturally diverse patients. Three items were reverse coded (numbers 6, 20, and 23 in Table 1) to associate higher points with better practice to be consistent with the rest of the items. The questionnaire and its 3 subscales were validated by 2 content experts who are authors of this manuscript, and are also faculty who continue to deliver the studied multicultural curriculum. Pre questionnaires included an additional section to collect students’ demographic information, and based on these responses, students were categorized into age groups (initially 20-24, 25-29, and 30 or older; then dichotomized into 25-29 and all others), gender, immigration status (initially immigrant/child of an immigrant, grandchild of an immigrant, or none of the above; then dichotomized into within 3 generations of immigrants and not within 3 generations of immigrants), and race (white or non-white). All non-white students were collapsed into one group given small subsample sizes of particular racial groups (e.g., African Americans).

**Table 1:** Demographic characteristics of participants from AY2000/2001 to AY2010/2011 (n=1066)

Variables		n	%
Academic Year			
	2000-2001	112	10.5
	2001-2002	94	8.8
	2002-2003	101	9.5
	2003-2004	95	8.9
	2004-2005	73	6.8
	2005-2006	80	7.5
	2006-2007	102	9.6
	2007-2008	98	9.2
	2008-2009	96	9.0
	2009-2010	102	9.6
	2010-2011	113	10.6
Age			
	20-24	162	15.2
	25-29	672	63.0
	30+	166	15.6
	Missing	66	6.2
Race			
	White	728	68.3
	Non-White	183	17.7
	Multiple races	39	3.1
	Missing	116	10.9
Gender			
	Male	456	42.8
	Female	532	49.9
	Missing	78	7.3
	Immigrant /Child of immigrant	273	25.6
	Grandchild of Immigrant	208	19.5
	None of the above	503	47.2
	Missing	82	7.7

The reliability of the measures including change in Cronbach alpha if an item is deleted, inter-item correlation, and item-to-group correlations were analyzed to assess each question’s validity in the measure.

### Statistical analysis

Various analytical strategies were used. Descriptive analysis included frequencies and prevalence of demographic factors. Wilcoxon signed rank tests were implemented to compare the change in item ratings between the pre and post questionnaires. Means of rating scores were calculated for the overall questionnaire as well as for each subscale of knowledge, empathy, and interview skills. Paired t-tests analyses were conducted and effect sizes were computed using Cohen’s d to assess the change of composite pre and post mean scores. Furthermore, MANOVA analyses were used to determine if demographic factors affected students’ initial ratings and change in ratings. Statistical significance levels were set at α=0.05. All analyses were performed using SPSS 19.0, IBM Inc.This study received approval as an exemption for human studies from the Institutional Review Board and Human Subjects Committee at the studied institution.

## Results

### Instrument’s reliability and validity

Course evaluation and pre/post questionnaire were constructed, and reviewed for face and content validity, by 2 of the 4 authors who are experts in multiculturalism education at our institution. The course evaluation had excellent reliability (Cronbach alpha=0.98), strong average inter-item correlation (0.75), and significant item-to-group correlations (ranged from 0.74 to 0.90). The pre/post questionnaires had high overall reliability (Cronbach alpha= 0.78) which changed very slightly when an item was deleted (from 0.76 to 0.79). These questionnaires also had an adequate average inter-item correlation of 0.38 and satisfactory item-to-group correlations (ranged from 0.40 to 0.68), suggesting that no items needed to be excluded. Therefore, all 20 items in the course evaluation and 23 items in pre/post questionnaires were included in subsequent analyses.

### Course evaluation

During the 11 studied years, 883 students completed the course evaluations with a high overall mean rating of 3.08 with a standard deviation of 0.45. The subscale mean scores ranged from 3.03 to 3.30 suggesting that students generally evaluated this interclerkship and its components positively. Trends in rating scores given to three out of four aspects (effectiveness, overall experience, and usefulness of the workshop) of the course evaluation reflected that average ratings started relatively low at the beginning (2000-2001), then increased considerably until reaching a plateau (2007-2008), and stayed fairly stable thereafter. These rating increases demonstrated a clear uptrend (p<0.0001). In 2002-2003, there was a noticeable decrease in ratings in all areas. Small group leaders received consistently high ratings in all years, except for the decrease in 2002-2003.

Positive correlations between rating of leaders and rating of usefulness were observed (correlation coefficients ranged from 0.28 to 0.34, p<0.0001), signaling that leaders played an important role in students’ perception of the program.

### Pre and post questionnaires

A total of 1,010 and 1,020 participants responded to the pre and post surveys, respectively. Of them, 967 students returned both questionnaires and were therefore included in the analyses. The number of student respondents gradually increased over the years from 67 students in AY2000-2001 to 111 students in AY2010-2011, which reflected an overall increase in class size from 100 to 125 during that time period. Of those responding the demographic information collected in the pre survey indicated participants were more likely to be female (53.9%), white (80.1%), in the age group of 25-29 (67.4%), and most did not have an immigration background within three generations (51.3%), compared to 27.6% who were first or second generation immigrants and 21.1% from third generation immigrant families (Table 1). These characteristics remained consistent throughout the eleven academic years.For the item level comparisons shown in Table 1, the majority of items (19 out of 23) had statistically significant higher post interclerkship ratings compared to pre scores (Wilcoxon Sign Rank Z_value ranging from 7.08 to 19.07, p <0.05), implying that students’ empathy, knowledge, and interview skills benefited significantly from the program. Nine of the 19 statistically significant items had very strong magnitudes of change in levels of agreement. Scores for all three negatively worded items requiring reverse coding (see Table 2 for details) did not significantly change after the interclerkship, possibly due to students’ confusion regarding the wording of each of those items. The only other item that did not show a statistically significant change in pre to post ratings was item 19, ‘a negotiated plan of care with a patient is a key component to patient satisfaction and compliance,’ which received the highest agreement rating at baseline (mean=3.24), and did not improve significantly at the follow-up (mean=3.26).

**Table 2:** Pairwise comparison of item ratings from AY2000/2001 to AY2010/2011 (matched n=967)

Item		Matched n	Pre Mean (SD)	Post Mean (SD)	Wilcoxon signed ranks Z-value	p
1	I know how to develop trust with patients of different cultures (E)*	964	2.99 (0.45)	3.10 (0.37)	7.08	0.00^†^
2	I am aware of the hardships faced by many newcomers (E)	965	2.99 (0.56)	3.15 (0.45)	9.12	0.00^†^
3	I am likely to ask newcomer patients about the circumstances of leaving their homeland (E)	964	2.68 (0.63)	3.18 (0.48)	19.07	0.00^†^
4	I am likely to ask newcomers about how their lives have changed in the US (E)	963	2.77 (0.57)	3.17 (0.47)	17.50	0.00^†^
5	I have the training to treat patients of cultures different from my own (K)	962	2.66 (0.61)	3.02 (0.44)	16.06	0.00^†^
6	I am likely to feel unsympathetic to newcomers who do not try to learn English^R^ (E)	962	2.64 (0.85)	2.64 (0.91)	0.19	0.85
7	I am knowledgeable about cultural and racial differences in health practices and beliefs (K)*	958	2.67 (0.57)	2.99 (0.43)	15.22	0.00^†^
8	I am able to recognize cultural bias in healthcare delivery (E)	953	2.85 (0.49)	3.05 (0.39)	11.34	0.00^†^
9	I have an understanding of the attitudes of other cultures in this area to conventional and alternative medical treatments (K)	957	2.52 (0.56)	2.97 (0.45)	18.63	0.00^†^
10	In order to perform a culturally sensitive interview one must be familiar with the individual's cultural differences (K)	960	2.87 (0.57)	3.02 (0.58)	7.62	0.00^†^
11	Allowing a patient to answer an open-ended question without interruption is more likely to elicit the patient's chief concern (I)*	959	3.20 (0.53)	3.23 (0.49)	2.11	0.03^†^
12	When using non-professional interpreters, it is important to direct them in their appropriate role in the interview (I)	962	3.08 (0.45)	3.17 (0.45)	5.78	0.00^†^
13	Patients and interpreters should be allowed to speak about an issue without interruption (I)	954	2.82 (0.59)	2.94 (0.61)	6.38	0.00^†^
14	Using open-ended questions when interviewing patients is likely to shorten the length of the medical visit (I)	950	2.56 (0.66)	2.83 (0.66)	12.08	0.00^†^
15	I have the skills necessary to successfully elicit a patient's health beliefs even when cultural differences exist (I)	957	2.74 (0.51)	3.06 (0.39)	15.51	0.00^†^
16	I have the skills necessary to explain my interpretation of a patient's condition to them in a way that they understand even when there are cultural differences between us (I)	957	2.77 (0.51)	3.05 (0.40)	14.16	0.00^†^
17	I have the skills necessary to negotiate a work-up and treatment plan with a patient from a different culture (I)	958	2.81 (0.48)	3.04 (0.40)	12.34	0.00^†^
18	Eliciting a patient's health beliefs is a necessary component for achieving positive patient outcomes (I)	957	3.17 (0.45)	3.22 (0.44)	3.08	0.00^†^
19	A negotiated plan of care with a patient is a key component to patient satisfaction and compliance (I)	958	3.24 (0.45)	3.26 (0.46)	1.39	0.16
20	Health outcome disparities between African-Americans and white Americans are purely a function of differences in socio-economic status^R^(K)	951	2.64 (0.71)	2.59 (0.81)	1.72	0.08
21	Myocardial infarctions are misdiagnosed more often in African-Americans than in white Americans (K)	935	2.89 (0.50)	3.09 (0.50)	9.78	0.00^†^
22	White Americans are offered more pain control medication than are African-Americans (K)	937	3.00 (0.50)	3.19 (0.50)	9.77	0.00^†^
23	Invasive procedures such as cardiac catherization and CABG are offered more often to African-Americans than to white Americans^R^ (K)	935	2.60 (0.62)	2.57 (0.76)	0.86	0.39

* E: Empathy, K: Knowledge, I: Interview Skills; ^†^Statistically significant at α=0.05; ^R^ Reverse coded question.

When combining items into an overall mean score and into mean scores by 3 subscales (empathy, knowledge, and interview skills), results indicated statistically significant increases in all after the interclerkship experience (see Table 3). The overall mean score increased to 3.02 in the post questionnaire compared to 2.83 in the pre questionnaire (t_(966)_=26.8, p<0.0001) with a large effect size (Cohen d=0.82), indicating a substantial overall benefit to students. Significant increases were also found for mean empathy scores (3.05 post vs. 2.81 pre, t_(966)_ =21.2, p<0.0001) and knowledge scores (2.93 post vs. 2.73 pre, t_(966)_=21.6, p<0.0001) with large effect sizes (0.73 and 0.75, respectively). Additionally, the mean interview skills scores increased significantly (3.09 post vs. 2.93 pre, t_(966)_=20.3, p<0.0001) with a medium effect size of 0.56. These results suggested that an effective multiculturalism interclerkship can significantly improve students’ knowledge, empathy, and interview skills when working with culturally diverse patients.

**Table 3:** Subscale and overall ratings (matched n=967)

Subscales	No. items	n	Mean Score		Effect size	p
			Pre	Post	D	
Empathy	6	967	2.81	3.05	0.73	<0.0001^†^
Knowledge	8	966	2.73	2.93	0.75	<0.0001^†^
Interview	9	967	2.93	3.09	0.56	<0.0001^†^
Overall	23	967	2.83	3.02	0.82	<0.0001^†^

^†^denotes statistically significant difference

When assessing if student characteristics affect pre and post means, multivariate analyses (see Table 4) revealed that being non-white, coming from immigration background within three generations, and participating in the program in later years were more likely to be associated with higher initial overall ratings (F_(1,966)_=4.04, p=0.04; F_(1,966)_=8.26, p=0.004; and F_(1,966)_=5.52, p=0.019, respectively), while gender and age did not appear to affect students’ initial overall ratings. At the subscale level, empathy items received lower initial ratings among white students (p=0.002) and not within 3 immigration generations (p=0.002) (data not shown). Students who gave higher initial overall ratings and coming from later years were more likely to increase their overall rating post interclerkship. Additionally, students from within 3 generations of immigrant families showed a higher increase post vs. pre in overall rating (F_(1,966)_=4.37, p=0.037) (Table 5) as well as in empathy (F_(1,966)_=4.1, p=0.045) and interview (F_(1,966)_=4.28, p=0.039) but not in knowledge (p=2.242), indicating that they benefited more from the program in these statistically significant domains.

**Table 4:** Demographic factors associated with initial overall rating (n=967)

Variables	B	Std. Error	95% Confidence Interval for B		P
			lower	upper	
Constant	-8.281	4.754	-17.61	1.05	0.082
White Race	-0.044	0.022	-0.086	-0.001	0.044^†^
Age 25-29	0.019	0.013	-0.006	0.045	0.128
Not within 3 generations of immigrants	-0.029	0.010	-0.049	-0.009	0.004^†^
Male	0.005	0.015	-0.024	0.034	0.735
Year	0.006	0.002	0.001	0.010	0.019^†^

^†^denotes statistical significance

**Table 5:** Factors associated with increase in overall rating (n=967)

Model		Unstandardized coefficients		Standardized coefficients	t	p
		B	Std. Error	B		
1	(Constant)	-20.83	7.12		-2.922	0.004
	White Race	0.067	0.032	0.083	2.092	0.037^†^
	Age 25-29	-0.020	0.019	-0.036	-1.062	0.289
	Not within 3 generations of immigrants	0.005	0.015	0.013	0.325	0.745
	Male	-0.015	0.022	-0.023	-0.697	0.486
	Year	0.011	0.004	0.099	2.956	0.003^†^

^†^ denotes statistical significance

## Discussion

Given the increasing visibility and importance placed upon cultural competence, this study sought to assess whether a single day curriculum in a multiculturalism interclerkship can effectively improve students’ knowledge, skills, and empathy towards serving culturally diverse populations, and the results reveal significant growth in students’ cultural competence. Compared to pre scores, students’ post overall scores and scores in each of the three subscales for empathy, knowledge, and interview scores were significantly higher, with medium to large effect sizes. Furthermore, in the item level comparisons, nearly all items (19 out of 23) had statistically significant higher post ratings compared to pre ratings.Thus, the present findings suggest that short educational interventions from a single day can lead to significant improvements. Even short interventions, such as the multicultural interclerkship at this medical school, may serve as catalysts for student introspection and discussion about cultures less familiar to them with faculty, students, and patients. Prior research has indicated there is a relationship between the cultural competence of primary care providers and the clinics in which they work,^21^ so increasing multicultural exposure to all medical students may enhance the cultural competence of these future physicians which may help reduce healthcare disparities. Misra-Hebert^7^ suggested empathy can be improved through a multi-modal faculty development program, we suggest that similar strategies might be successfully employed within the instructional delivery to medical and other healthcare graduate programs. More specifically, medical students need continued exposure to ensure a keen and focused understanding of the impact their behavior as future physicians will have on closing the growing health disparity gap.However, some student characteristics may be associated with greater initial ratings and growth in cultural competence from educational interventions. As indicated by the results of pre scores, students’ race, immigration background, and the year of the interclerkship significantly affected students’ initial cultural competence, also referred to as cultural humility, before the interclerkship. Because students’ pre scores significantly and positively predicted post scores, these factors also indirectly affected post scores. Nonetheless, when controlling for pre scores, immigrant background and year were still significantly associated with post scores. Not only did students with more recent immigrant backgrounds and those participating in the later years of this educational experience start with greater cultural competence, they also gained more during the interclerkship, perhaps reflecting greater receptivity to multicultural curricula. Nunes^6^ suggested that changes be introduced that encouraged support of students academically and emotionally throughout their education. We also believe the educational value of this support would be beneficial to all healthcare students, but especially important to those whose initial ratings are lower than their peers at the start of their educational journey. This is supported by our findings that race and immigration history may play a role in level of receptivity to curriculum focused on increasing cultural humility. In other words, while this exposure would most likely benefit all students, perhaps the most in need may benefit the most. Further research is warranted in this area.

The year may have been significant due to the increasing level of sophistication of incoming students. In other words, the trend in more recent cohorts may reflect increased exposure to multicultural education and/or experiences prior to medical school, such as the increased emphasis of multicultural curriculum in K-16 education. Students with more recent immigrant backgrounds may have been more receptive to the messages in the multiculturalism curriculum due to their personal and familial experiences. Furthermore, belonging to any racial minority group may influence receptivity to a multicultural interclerkship as highlighted by this study finding that initial empathy ratings were lower among white students who were not within 3 immigration generations. Future research could assess other factors that may influence initial levels and growth in learning of cultural humility/cultural competence, the results of which could be used to enrich all students’ learning. We did not identify any published results in this specific area of research.While we did not find gender and age to be statistically significant factors in this study, this highlights similar findings in published data. These 2 specific factors and cultural competence, with empathy as a focus, has produced inconsistent findings to date.^6^^-^^9^ We suggest further research is warranted as the more academic leaders understand the role age and gender plays, the better the curriculum can be tailored to ensure training in cultural humility is delivered in a meaningful manner. Educators want to use instructional time wisely given the importance cultural competence has on skills and abilities of future healthcare professionals, and the indirect impact their attitude and behavior has on healthcare disparities.Notably, students generally evaluated this interclerkship and its components very positively, which may be related to its effectiveness in improving cultural competence. The results indicated that student ratings of course evaluations increased over time until reaching a plateau (perhaps reflecting stabilization of instructional delivery). Therefore, multicultural curricula at other institutions may also experience lower evaluations when first implemented but likely could increase over time as content, exercises, and teaching methods are refined.We believe our findings and those of previously cited studies highlight the need for future retrospective reviews of actual practice locations of healthcare professionals as well as inquiries into their attitudes about decreasing healthcare disparities of their patients. Researchers need to assess what healthcare professionals know, believe, and practice, in order to have an impact on this multi-layered global topic. Future research findings could then inform the curricula connected to cultural competence. The role of healthcare professionals in reducing healthcare disparities cannot be emphasized enough.

### Limitations of study

While this research study included data spanning 11 years, thereby providing a large sample size increasing statistical power and the ability to implement subset analyses, there are several limitations that could serve as catalysts for future research. First, participating students were from a single medical school and in their third year, which may have influenced the results. Incorporating multicultural curricula in different school environments or at different points in medical school education could influence the efficacy. Future research should assess the effectiveness of multicultural curricula at different institutions and across different levels of education. Second, the research did not evaluate students’ actual skills or knowledge, but rather measured students’ perceptions of their own skills and knowledge through self-reported data. Thus, this research has similar limitations to all research that relies on self-reported data. Additionally, the impact of recall bias on the self-reported data is diminished due to the large magnitude of the association between pre and post ratings (overall effect size =0.82), the strong statistical significance (p <0.0001), and the consistent associations across different years and subscales. Avenues for future research include using direct measures of students’ knowledge and skills. Finally, because the post responses were collected immediately at the end of the interclerkship, the longer term effects and the durability of the gains cannot be assessed. We are currently designing a research project to assess the long-term effects of this interclerkship.

## Conclusion

In conclusion, the multicultural interclerkship in this study appeared to be beneficial to students. By increasing their exposure and interaction with diverse patients, their knowledge, attitude, and skills were increased and expanded in a positive manner. In the introduction, the aforementioned joint panel described cultural competence in the broader context of diversity and inclusion as “the active, intentional, and ongoing engagement with diversity to increase one’s awareness, content knowledge, cognitive sophistication, and empathic understanding of the complex ways individuals interact within systems and institutions”.^3^ Given this broad definition, it is clear that medical school curriculum must help students reflect on their attitudes and assumptions about culture and ethnicity in general. In addition, medical educators must also push students to realize the depth to which they must focus on their biases in order to push beyond into unchartered and often new realities. While this introspection must occur in a “safe” learning environment, it is not enough to present information about culture groups, students must observe and participate in “best practice” scenarios in order to move beyond broadly defined ethnic and cultural issues connected to various populations. We believe it would be extremely important to interact with patient cultures different from student’s own to better develop cultural humility. As health disparities among underrepresented populations become an increasing problem in the world today, interprofessional education within and across medicine, nursing, and health sciences may provide a vehicle for cross-cultural training as a strategy to minimize future health disparities.^16^ Given its potential to indirectly impact global healthcare delivery, perhaps an increase in focus at the international level within and across medical schools could greatly improve healthcare disparatives. This could include a more streamlined approach to new and/or existing reciprocal arrangements between all North American and international healthcare professional institutions. The findings from this study might inform those who are also interested in enhancing this important competence within any and all medical schools. This is especially true given the increasing scrutiny this global topic is receiving within and across all healthcare professions today.

### Conflict of Interest

The authors declare that they have no conflict of interest.
